# Preparation of Isotropic Carbon Fibers from Kerosene-Purified Coal Tar Pitch by Co-Carbonization with Pyrolysis Fuel Oil

**DOI:** 10.3390/ma14216280

**Published:** 2021-10-21

**Authors:** Seon Ho Lee, Song Mi Lee, Seungjoo Park, Seong-Ho Yoon, Haksoo Han, Doo-Hwan Jung

**Affiliations:** 1 Fuel Cell Research Center, Korea Institute of Energy Research (KIER), Daejeon 34129, Korea; pirsys@kier.re.kr (S.H.L.); songmi@kier.re.kr (S.M.L.); tmdwn0903@kier.re.kr (S.P.); 2 Department of Chemical and Biomolecular Engineering, College of Engineering, Yonsei University, Seoul 03722, Korea; 3 Department of Advanced Energy Technology, University of Science and Technology, Daejeon 34113, Korea; 4 Institute for Materials Chemistry and Engineering, Kyushu University, 6-1 Kasuga koen, Kasuga-shi 816-8580, Fukuoka, Japan; yoon@cm.kyushu-u.ac.jp

**Keywords:** coal tar pitch extract, pyrolysis fuel oil, centrifugation, spinnable pitch, isotropic carbon fiber

## Abstract

An inexpensive and general-purpose carbon fiber was prepared using coal tar pitch. In contrast to the solvent extraction process employing expensive solvents, a low-cost centrifugal separation method facilitated the reduction of loss due to the pitch purification and an overall yield increase. The coal tar pitch purified by centrifugation and subsequently co-carbonized with pyrolysis fuel oil improved in spinnability. Moreover, the resulting spinnable pitch had a softening point of 250 °C. The obtained carbon fibers were heat-treated at 1000 °C for 5 min, resulting in a tensile strength of approximately 1000 MPa and an average diameter of 9 μm. In this study, we present an effective method for obtaining low-cost general-purpose isotropic carbon fibers.

## 1. Introduction

Fuel economy is in high demand owing to the depletion of petroleum resources and environmental problems caused by large amounts of fine dust emissions. Therefore, reducing vehicle weight, improving the engine efficiency, and enhancing the energy transfer efficiency are vital for decreasing carbon emissions and boosting fuel economy [1,2,3]. In particular, a 10% reduction in vehicle weight can result in a ~7% fuel economy improvement [4]. Therefore, the development of fuel-saving technologies enabling vehicle weight reduction has become essential. Lightening the weight of the vehicle body structure can be achieved by using high-tensile steel and aluminum alloys, but the use of carbon fiber-reinforced plastics (CFRP) enables the production of the best-performing lightweight components for an automotive. The use of CFRP alone in the body structure causes a weight reduction of ~50%. Carbon fiber, the main component of CFRP, is used by itself, or in combination with other materials, to form diverse composites, such as carbon–carbon (C/C), plastic, metal, and ceramic composites [5,6,7].

The pitch-based carbon fibers can be anisotropic or isotropic, depending on whether the measured properties are direction-dependent or not, respectively. The isotropic pitch-based carbon fibers are inexpensive and highly performant, compared to the anisotropic pitch-based carbon fibers; therefore, they are referred to as general-purpose carbon fibers. Produced with the melt-blown method, they serve as activated fibers for high-temperature insulators and filters [8,9,10]. Coal tar pitch, a raw material for pitch-based carbon fibers, is obtained by distilling coal tar, a by-product obtained during the coke manufacturing process in a steel mill. Compared to other fibers such as those based on polyacrylonitrile (PAN), pitch-based carbon fibers show high performance when prepared from well-refined pitch. Furthermore, liquid crystal pitch is the raw material used for anisotropic pitch-based carbon fibers with sufficient tensile strength and modulus, used in the aerospace industry. To obtain liquid crystal pitch from petroleum and coal tar, a complex pretreatment process, including quinoline insoluble matter removal and a hydrogenation process, is required. The resulting production and material costs are consequently high. Therefore, the development of new isotropic carbon fibers should take into account low-cost mass-production and achievement of all the lightweight CFRP physical properties [11,12,13,14,15].

Co-carbonization is the process of mixing two different materials, and applying a thermosetting procedure to allow carbonization. Nabil et al. obtained high-quality fibers by polymerizing poly acrylonitrile resin and phenolic resin [16]. Lin et al. produced isotropic spinnable pitch by co-carbonizing coal tar pitch in bio-asphalt [17]. Additionally, Yang et al. produced high-quality carbon fibers by co-carbonizing ethylene bottom oil (EBO) in coal tar [18]. Co-carbonization largely contributes to the high quality of the obtained fibers. During this process, the free space within the polycyclic pitch structure is filled with relatively low-molecular-weight molecules, which avoid crack formation due to thermal contraction caused by stabilization and further carbonization. Coal tar pitch has a higher aromaticity than petroleum residues, mainly containing aliphatic components, so a high yield is expected. Conversely, the petroleum residue is characterized by high spinnability. Co-carbonization is therefore an effective procedure to obtain a pitch with suitable properties and excellent spinnability [1,11,17,18].

In this study, we aimed to obtain a spinnable pitch for the preparation of isotropic carbon fibers. Coal tar pitch was first centrifuged with the low-cost Kerosene method, then co-carbonized with pyrolysis fuel oil (PFO). By modulating the mixing ratio, we determined the conditions leading to optimal physical properties and cost reduction. We performed elemental and structural analysis of the co-carbonized pitch, and the prepared carbon fibers were subsequently subjected to surface observation, component analysis, and mechanical properties’ measurements.

## 2. Experimental

### 2.1. Materials

Coal tar pitch (SP 30 °C, Posco Chemical Co., Pohang, Korea) was used as the raw material for the preparation of spinnable pitch, the precursor of the final isotropic carbon fibers. Additionally, cross-linked molecules were formed by the co-carbonization of pyrolysis fuel oil (PFO, GS-Caltex Co., Seoul, Korea) and coal tar pitch. The properties of the raw material are listed in Table 1. Two methods have been used to remove quinoline insoluble compounds, that adversely affect carbon fiber production. First, the coal tar pitch was dissolved in tetrahydrofuran (THF) by solvent extraction, and the precipitated insoluble particles were filtered out using a vacuum pump. Alternatively, the Centrifuge Kerosene Equivalent method, consisting of melting coal tar pitch in an oven at 150 °C and purifying it to a liquid state, was employed to remove the insoluble precipitate by centrifugation.

### 2.2. Preparation of the Spinnable Pitch

**Figure 1:**
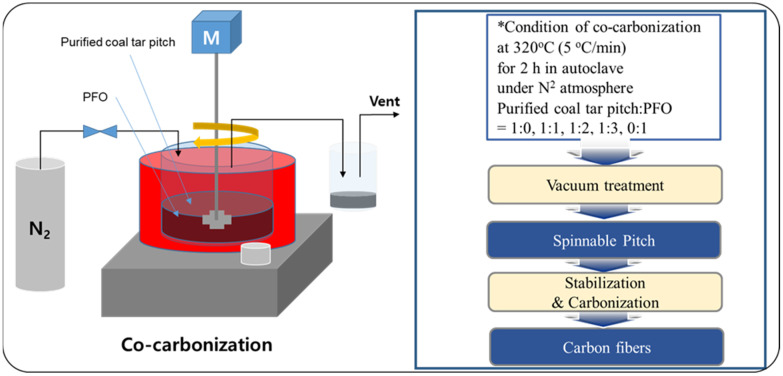
Illustration of the production of isotropic carbon fiber by co-carbonization.

The spinnable pitch was prepared via the co-carbonization method of coal tar pitch and PFO, as shown in Figure 1. PFO was added to the coal tar pitch, previously purified by centrifugation, to promote spinnability. Purified coal tar pitch (50 g) was mixed with PFO according to the weight ratios 1:0, 1:1, 1:2, 1:3, and 0:1. To allow a complete mix, PFO and the coal tar pitch were stirred and heated at 100 °C in an autoclave, under nitrogen atmosphere. Thereafter, the temperature was raised to 320 °C at a rate of 5 °C/min, followed by heat treatment under pressure for 2 h, to reform the product. Upon heat treatment completion and cooling down, the modified pitch was collected. To increase the softening point of the prepared pitch, the low-molecular-weight volatile matter generated in the gaseous form was removed by a vacuum heat treatment with a vacuum pump and a cooling trap. Vacuum heat treatment was performed for 10 min, raising the temperature to 300 °C at a rate of 10 °C/min. The obtained spinnable pitch showed a softening point of 250 °C. The thus-prepared isotropic spinnable pitches were named C100, C50P50, C33P67, C25P75, and P100 according to the co-carbonization ratio of purified coal tar pitch to PFO.

### 2.3. Preparation of Isotropic Carbon Fibers

The prepared spinnable pitch was spun into fibers through a melt spinning method using a spinning apparatus with a spinneret of 0.2 mm diameter and length/diameter = 2. Pitch fibers were spun using a laboratory-type monofilament melt spinning machine as a general melt spinning method for the preparation of pitch-based carbon fibers [1,2,11]. Spinning experiments were performed under 0.2 MPa nitrogen pressure at a temperature of SP + 50 °C. Isotropic carbon fibers were obtained through stabilization and carbonization of pitch fibers. Pitch fibers were first stabilized in a muffle furnace for 1 h with a heating rate of 0.5 °C and air flow rate of 200 mL/min. Notably, the section was 20 °C higher than the softening point. Following this, the stabilized fibers were carbonized in a tubular furnace to 1000 °C for 5 min, with a heating rate of 5 °C/min and 200 mL/min of nitrogen.

### 2.4. Analysis

The determination of carbon, hydrogen, nitrogen, and sulfur content in the samples was achieved with an elemental analyzer (MT-5 CHN Corder, Yanako Co., Tokyo, Japan) and a sulfur analyzer (LECO 744 series, LECO Co., Ann Arbor, MI, USA). The elemental analysis revealed the changes in the physical properties of the raw material and the pitch manufactured under various conditions. The oxygen content (wt.%) was calculated by Equation (1) [19]:

O = 100 − C − H − N − S(1)

The molecular weight distribution of the soft pitch was determined using TOF-MS (JMS-S3000, JEOL Co., Tokyo, Japan). A portion of 0.5 mL of sample dissolved in THF was analyzed, upon drying. The operating parameters were controlled with a laser intensity of 40–60% and a delay time of 280 ns. The chemical structure investigation, with an emphasis on the pitch fiber stability, was performed by using a Bruker VERTEX 80v Microscope Vacuum Infrared Spectrometer (IR) in transmittance mode. Fourier transformed (FT) IR spectra were obtained with the KBr method using pellets (100 mg) with lyophilized samples (1–2 mg dry solids). The structures of the samples were characterized by X-ray diffraction (XRD; D/MAX-2500PC, Rigaku Co., Tokyo, Japan), and the patterns were obtained to investigate the properties of the carbon support and Pt. Scans were carried out at 5°/min for 2 values between 10° and 90°. A scanning electron microscope (SEM; S-3500N, Hitachi, Tokyo, Japan) was used to measure the diameter of the produced carbon fibers, whose surfaces were coated with platinum. The tensile tests were determined using the ASTM standards (ASTM C1557-14). For each sample, five specimens were prepared, and the average value of the measurement was considered. The cross-sectional area of the fiber was investigated with SEM after attaching the separated fiber to the tensile-test fiber-mounting paper.

## 3. Results and Discussion

### 3.1. Purification of Coal Tar Pitch

Coal tar pitch with a softening point of 30 °C and a C/H ratio of 1.5 was purified using two methods: solvent extraction and centrifugation. For the solvent extraction method, THF was used as a solvent; upon dissolution, the sample was filtered under vacuum. For the centrifugation method, kerosene was heated at 150 °C; afterwards, the solid content was separated and extracted, and finally filtrated and purified. Table 2 shows yield and softening point correlation to the reaction temperature in the heat treatment process of the purified coal tar pitch using the two different methods. In the case of centrifugation, both the softening point and the yield improved with the reaction temperature increase. Generally, coal tar pitch is dissolved by THF, while kerosene generates a significant amount of insoluble matter at 20 °C. However, the pitch can be dissolved even in low-cost solvents, such as kerosene, when properly increasing the temperature until 150 °C. Moreover, kerosene can be used to purify the ash component, which is unnecessary for the production of spinnable pitch [20]. In conclusion, through centrifugation, the yield is greatly improved, and the softening point can be raised even for cost-effective low-temperature treatments.

### 3.2. Characteristics of Spinnable Pitch from Purified Coal Tar Pitch and PFO

**Figure 2:**
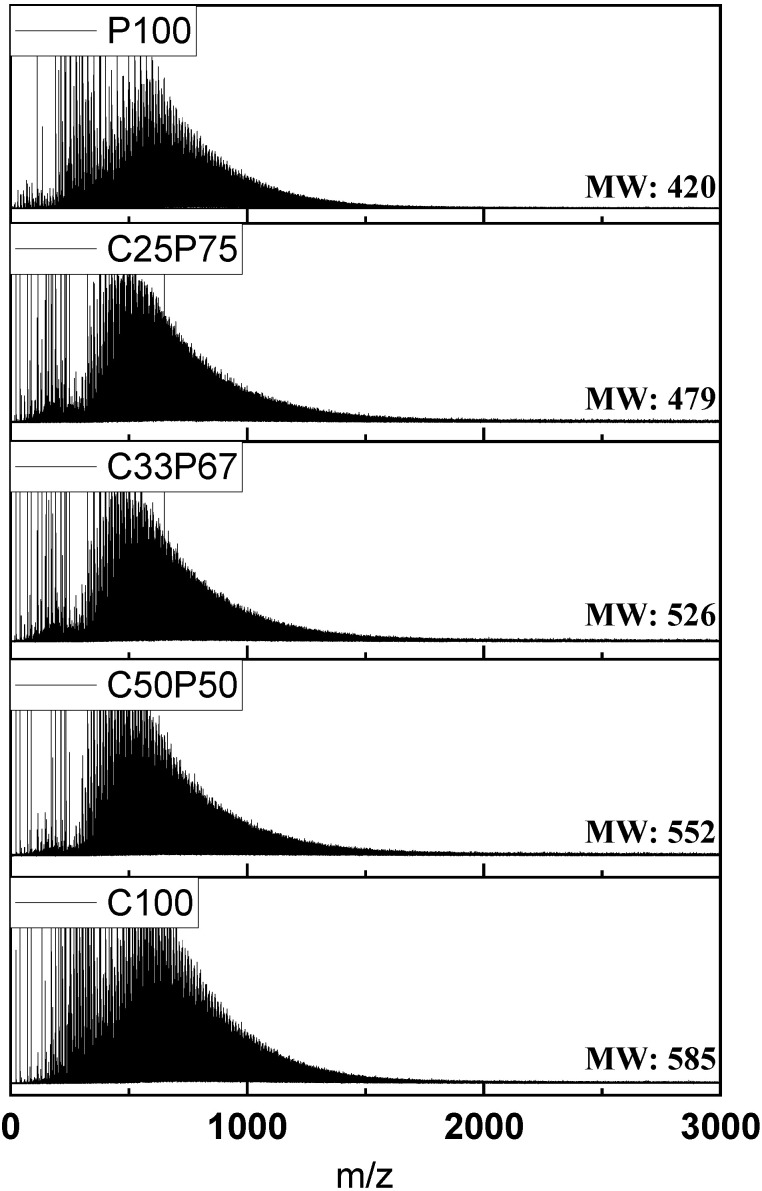
MALDI TOF-MS spectra of spinnable pitches prepared by co-carbonization of purified coal tar pitch and PFO in various ratios.

When the purified coal tar pitch is heated to allow polymerization, radicals are formed, due to hydrogen desorption. The formed radicals are combined with other surrounding pitches to form linear pitches [2]. The advantage of producing spinnable pitch using the co-carbonization method was that the addition of relatively low-molecular-weight PFO filling the free space between the polymers led to a stable structure [11]. Moreover, thanks to the stable structure, the thus-prepared spun fibers were expected to suppress the formation of cracks during the heat shrinkage in the subsequent stabilization and carbonization processes, contributing to the improvement of the mechanical properties [21]. Table 3 reports the elemental analysis results for each mixing ratio obtained with the co-carbonization method of purified coal tar pitch by kerosene centrifugation. The mixing ratio considerably affected the content of N, S, and O. Specifically, the intrinsic PFO properties neutralized the coal tar pitch through the co-carbonization process, leading to a decrease of the S and O content. Figure 2 shows the MALDI TOF-MS spectrum of the co-carbonization method at different mixing ratios. The molecular distribution was broad (100–1500 *m*/*z*). Among all the samples, the average molecular weight of C50P50 (*m*/*z* = 552) was the highest, while for C25P75 (*m*/*z* = 479) it was the lowest. In particular, the distribution below *m*/*z* = 300 was considerably affected by PFO content and, ultimately, by the co-carbonization process.

The pitch for carbon fiber production should have good spinning properties and uniform viscosity at the operating temperature. To this end, appropriate molecular weight and limited molecular weight distribution are required [2]. Systems with too-low molecular weight undergo ruptures during the spinning, while non-uniform molecular weight distribution results in irregular fiber thickness. Additionally, for samples containing liquid crystals in the isotropic phase, the narrow molecular weight distribution is particularly important, since the increase of viscosity may affect the spinning uniformity [11].

**Figure 3:**
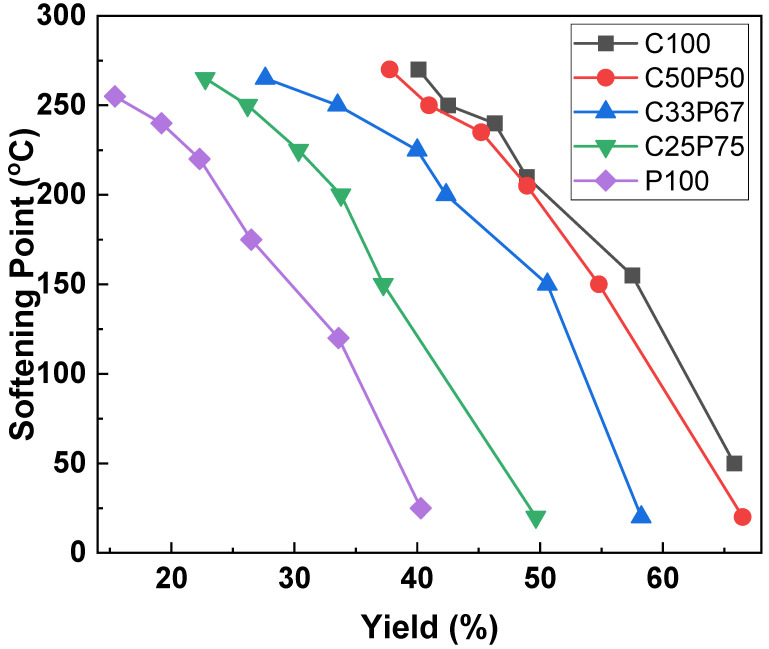
Softening point and yield changes of spinnable pitches prepared by co-carbonization of purified coal tar pitch and PFO in various ratios.

In this study, the softening point was controlled, and the spinnability was increased through a reforming process that extracted small molecules in a vacuum. The physical properties of the precursor pitch were controlled by adjusting heat treatment temperature, reaction time, and speed. Figure 3 shows the change in yield and softening point as a result of vacuum heat treatment, by adding different PFO ratios to the purified coal tar pitch. Overall, the yield decreased with the heat, and the softening point increased accordingly. The yield of the C100 using a purified coal tar pitch alone was the best, but the spinnability was not easily formed and was frequently broken. In the case of the P100 using PFO alone, the yield was the lowest, but the spinnable structure was relatively easy. However, at a high temperature of more than 240 °C of the softening point, it is difficult to maintain spinnability and it frequently breaks, much like purified coal tar pitch alone. Purified coal tar pitch has a problem in that it generates an intermediate phase in the process of raising its softening point for spinning, resulting in a significant decrease in spinnability. Thus, in the case of a mixed pitch of coal tar and PFO, an interaction between the two components occurs that offsets the properties of coal tar pitch that inhibit spinnability through the inhibitory effect of mesophase development. The physical properties of the fibers depended on the purified coal tar pitch and PFO contents [2,11,18]. PFO content and yield loss were directly correlated. In particular, for higher treatment temperatures, the yield decrease was substantial. When the softening point exceeded 260 °C, it had a heterogeneous phase as a whole, and because of this, the elongation due to the heterogeneous material was sharply lowered, resulting in an inappropriate pitch for spinning. Therefore, the best softening point of the spinnable pitch, obtained under each set of conditions, was 250 °C.

### 3.3. Characteristics of Pitch Fibers from Purified Coal Tar Pitch and PFO

A carbon fiber was spun at a winding speed (500 RPM) using a melt-spinning method with an appropriate spinning pitch. The change in the softening point during spinning is generally used to identify thermal characteristics [19]. The results confirmed that the PFO content was inversely related to the thermal stability of the pitch. In particular, in the case of C25P75, spun pitch fibers were easily entangled in the winder, and the shape of the fibers was easily distorted. Furthermore, fiber breakage did not occur in C25P75 and C33P67, but did occur in C50P50 (it was broken 5 times per 3 min), indicating a low spinnability for this sample. The precursor pitch produced only with coal-based soft pitch, without co-carbonization, did not radiate smoothly because there was no spinnability. The diameter of the spun fiber was varied from 7 to 15 μm. It was noticed that the coal tar pitch purification and double the weight of PFO were very effective for spinning.

**Figure 4:**
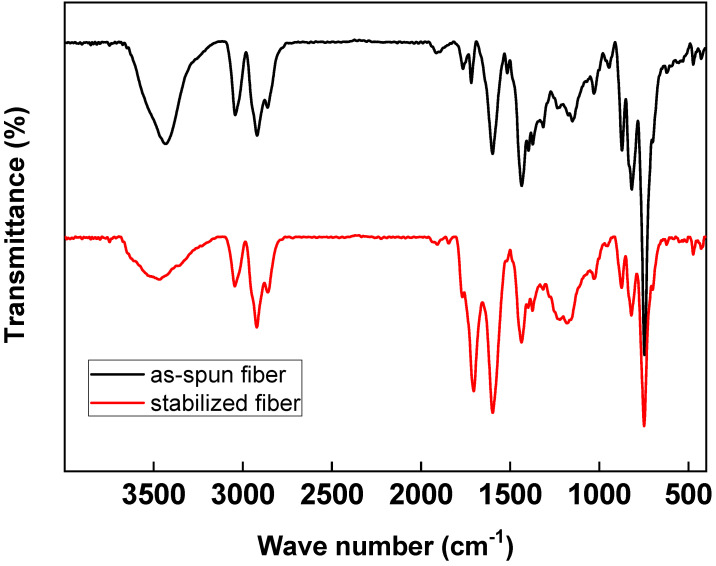
IR peaks of as-spun fiber and stabilized fiber from C33P67 in an air atmosphere at 270 °C for 1 h.

The spun fibers were stabilized in an oxygen atmosphere at a softening point of +20 °C. Through the stabilization process of pitch fibers, chemical and physical changes such as oxidation, dehydration, condensation, and crosslinking occur [22]. Thus, the shape of the fibers can be retained during carbonization. Figure 4 shows the results of the FT-IR analysis of spun fibers and stabilized fibers from C33P67 at 270 °C in the air for 1 h. Generally, the peak of pitch at 1670–1780 cm^−1^ was assigned to the C=O (carboxyl) group, and at 1600 cm^−1^, the double-bond region of aromatic C appears. The peaks at 3150–2990 cm^−1^ were assigned to O–H stretching and aromatic C–H stretching, 2990–2850 cm^−1^ was attributed to aliphatic C–H stretching, and 1400 cm^−1^ belonged to methylene C–H in-plane bending. Besides, the aromatic C–H out-of-plane bonding appeared at 900–700 cm^−1^ [11,23]. The stabilization process is also important in terms of carbon fiber quality, because heating the stabilized fibers to high temperatures during carbonization can cause the decomposition of oxygenated aliphatic groups in the pitch molecules, resulting in defects [2]. The –OH peak at 3450 cm^−1^ was reduced during the stabilization process, while the elastic absorption band increased at 1700 cm^−1^ related to the carbonyl group (C=O) rather than the spun fibers [23,24,25,26,27]. Additionally, the absorption band related to ether bonds (1260 cm^−1^) increased as well, indicating polymer dehydrogenation and oxygen penetration into the spinning fiber [11]. Therefore, oxygen was injected in the material during the stabilization process, leading to the generation of methylene groups connecting the naphthene rings and allyl aggregates at the fiber surface. Infusibilization was achieved by ether crosslinking [2,23].

The spun fibers were carbonized by heat treatment at 1000 °C after stabilization. In the fibers containing carbonyl as a functional group after stabilization, CO was desorbed, and radicals were formed due to the high temperatures of the carbonization process. These radicals shared their free electrons to form new bonds. In the case of aromatic rings, the double-bond π electrons are transferred, and radicals are generated. The process is repeated, and new bonds are formed. As this process proceeds, the hydrogen connected to the carbon is released, and another aromatic ring substituted with it can be bonded to the vacant position, resulting in final carbonization [22,28].

**Figure 5:**
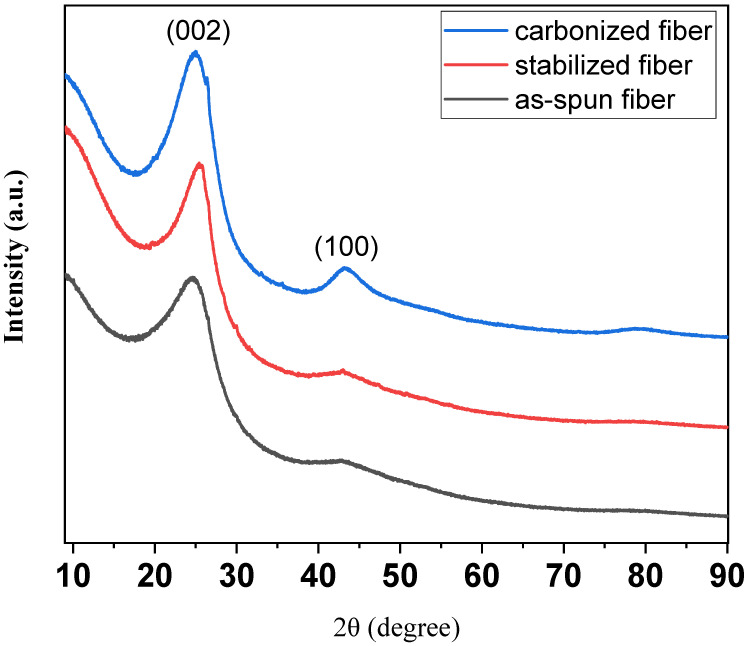
XRD patterns of as-spun fiber, stabilized fiber, and carbonized fiber from C33P67.

Figure 5 shows the XRD patterns of as-spun fiber, stabilized fiber, and carbonized fiber from C33P67. In the fiber prepared through the entire process, a (002) peak of the graphite structure appeared as a whole, and it can be confirmed that a (100) peak corresponding to the interlayer thickness of graphite was developed through the carbonization process at 1000 °C [29]. According to Bragg’s law, the interlayer spacing decreased from 0.3426 to 0.3307 nm due to structural development through the carbonization process. As a result, it was determined that the preparation of isotropic carbon fibers with excellent physical properties was possible [30].

### 3.4. Characteristics of Isotropic Carbon Fibers from Purified Coal Tar Pitch and PFO

**Figure 6:**
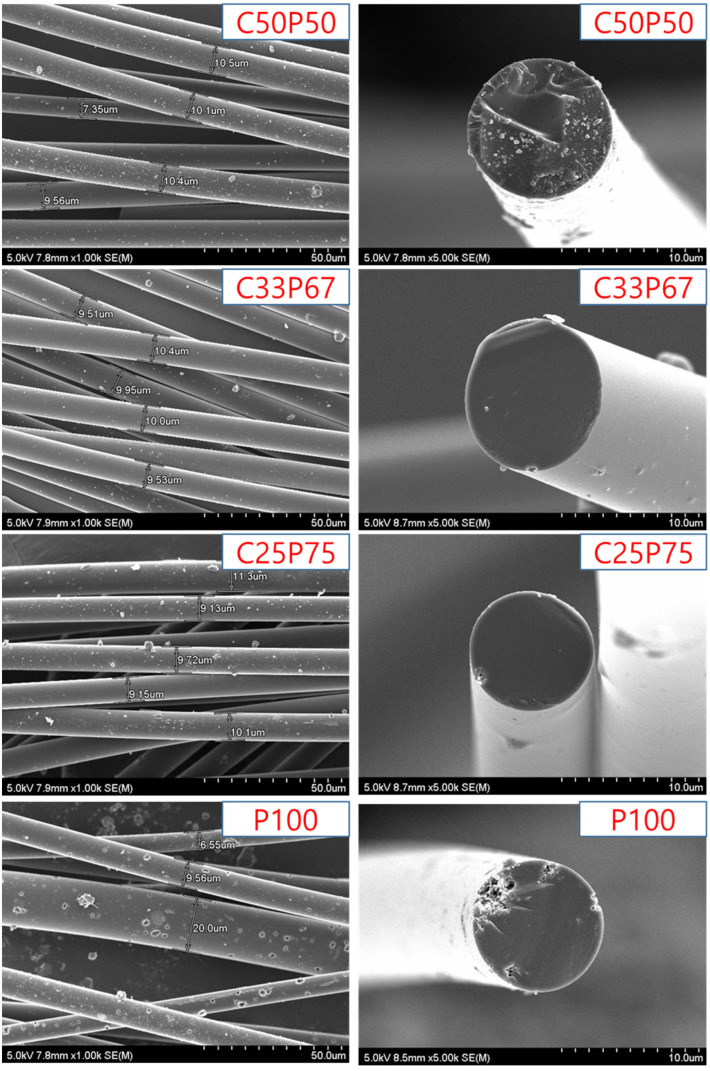
SEM analysis of isotropic carbon fibers prepared by co-carbonization of purified coal tar pitch and PFO in various ratios.

Figure 6 shows the surface and cross-section of the carbon fibers obtained under each set of conditions.

The result shows that the thickness of each fiber was uniform, equal to 9 μm on average. It can be seen that the obtained fibers had a smooth cross-section, an isotropic structure, and the shape of the surface was flatten and uniform. As shown in Table 4, C100 was not smoothly prepared for the spinnable pitch. On the other hand, many cracks were found on the surface and cross-section of the fiber spun with P100, and it can be confirmed that a complete isotropic pitch fiber was not formed. The mixing of PFO and purified coal tar pitch inhibits the formation of mesophases, leading to complete isotropy of the resulting pitch mixture. In addition, the fibers were uneven to such an extent that diameters exceeding 20 μm were often found. Therefore, for the development of general-purpose carbon fiber, it is very important to mix purified coal tar pitch and PFO in an appropriate ratio. However, the cross-section of the fibers spun with C50P50 was non-uniform and cracks were observed, because the PFO content was insufficient during the co-carbonization process and the preparation of complete isotropic pitch failed [31,32]. As a result of surface observation, no cracks were observed in C33P67, and it was confirmed that complete isotropy was achieved. Co-carbonization of an appropriate amount of purified coal tar and PFO has been shown to have a significant effect on the surface properties of the fibers.

**Figure 7:**
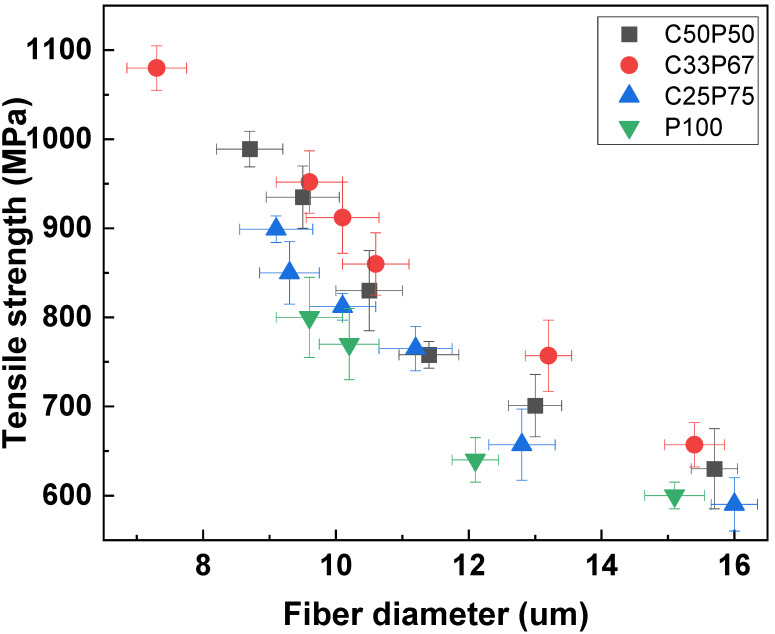
Tensile strength of isotropic carbon fibers prepared by co-carbonization of purified coal tar pitch and PFO in various ratios.

Figure 7 shows the tensile strength of the carbon fibers obtained at different conditions. As shown in Figure 3, The mixing of PFO and purified coal tar pitch, which have different properties, inhibits the formation of intermediate phases, leading to complete isotropy of the resulting pitch mixture. Therefore, the physical properties of the fibers depended on the purified coal tar pitch and PFO contents [2,11,18,31]. Figure 5 and Figure 6 confirm the correlation between the surface and cross-sectional shape of the fibers and their tensile strength. It shows that the mechanical properties of typical pitch-based carbon fibers generally increase with decreasing fiber diameter. Based on their rheological properties, linear compounds of spun pitch produce more highly oriented structures through stretching in the process of spinning into pitch fibers. In addition, the linearity of the molecules in the prepared pitch fibers facilitates the preparation of more homogeneous fibers through stabilization and carbonization, which affects the mechanical properties [31,33]. The higher the content of purified coal tar pitch, the better the tensile strength compared to the thickness of the fiber. As described above, PFO is easily observed as a heterogeneous phase at a softening point at a relatively high temperature due to its thermal characteristics, so that it cannot maintain spinnability and a lot of cracks occur. The addition of PFO has the advantage of increasing spinnability, and at the same time, the disadvantage of causing cracks due to the emission of volatile matter. For this reason, co-carbonization of an appropriate mixing ratio is necessary. Table 5 reports the mechanical properties of isotropic pitch-based carbon fibers prepared by various methods and raw materials. The isotropic pitch-based carbon fibers of C33P67 have a uniform and smooth surface, with an average diameter of 9 μm, tensile strength of 1000 MPa, modulus of 65.6 GPa, and elongation of 2.85%. On the other hand, the isotropic pitch-based carbon fibers of C25P75 have a few small pores throughout the surface, with an average diameter of 10 μm, a tensile strength of 820 MPa, a modulus of 37.4 GPa, and elongation of 1.85%. This can be attributed to the release of volatiles during carbonization. These defects reduce the mechanical properties of carbon fibers. Similarly, the isotropic pitch-based carbon fibers of P100 have very large cracks across the surface and cross-section, with an average diameter of 11 μm, tensile strength of 700 MPa, modulus of 22.5 GPa, and elongation of 1.65%. The presence or absence of cracks on the surface and cross-section of carbon fiber has been shown to affect tensile strength. Isotropic pitch-based carbon fiber prepared by co-carbonizing purified coal tar pitch and PFO showed higher tensile strength than PFO alone. These properties were considerably better than those measured for the isotropic pitch-based carbon fibers prepared by other methods and materials [34,35,36,37,38,39,40,41]. However, the mechanical properties of hyper-coal-based carbon fibers using expensive solvents were relatively good [19,42]. We therefore demonstrated that the co-carbonization of purified coal tar pitch and PFO is an effective method for obtaining a precursor of isotropic pitch-based carbon fibers with satisfactory mechanical properties at reduced costs.

## 4. Conclusions

Coal tar pitch, which is easy to transport, was used to prepare inexpensive and general-purpose carbon fibers. Instead of the solvent extraction method using expensive solvents, the low-cost centrifugal separation method was employed to reduce the loss due to the purification of the pitch, thereby significantly increasing the yield. The spinnability was very low when the purified coal tar pitch was used alone. To improve this process, co-carbonization was performed using PFO. The PFO content was controlled to considerably improve the cracks on the fiber surface; consequently, spinnability and mechanical properties improved. When the PFO content was double the coal tar pitch content, isotropic carbon fibers, with an average diameter of 9 μm, tensile strength of 1000 MPa, modulus of 65.6 GPa, and elongation of 2.85%, were obtained. Compared to previously reported isotropic pitch-based carbon fibers, the obtained isotropic carbon fibers were characterized by a thinner diameter and higher tensile strength. The method adopted in this study turned out to be effective for obtaining low-cost, general-purpose isotropic carbon fibers.

## Figures and Tables

**Table 1:** Elemental analysis of raw materials.

Elemental Analysis					
	C (wt%)	H (wt%)	N (wt%)	O (wt%)	S (wt%)
Coal tar pitch	86.9	6.1	1.5	4.9	0.5
PFO	90.3	7.9	1.6	0.1	0.1

**Table 2:** The analysis results of the yield and softening point according to solvent extraction and centrifugation.

Type	Vacuum Heat Treatment Temp. (°C)	Maintain (min)	Final Yield (%) (Pristine to Final Pitch)	Softening Point (°C)
Centrifugation (Kerosene)	200	10	81.1	100
	250	10	74.6	160
	350	10	52.4	250
Solvent extraction (THF)	200	10	64.8	80
	250	10	55.1	140
	350	10	40.3	190
	360	5	33.1	250

**Table 3:** Elemental analyses of spinnable pitches prepared by co-carbonization of purified coal tar pitch and PFO in various ratios.

Sample Name	Ratio (wt.%)		Elemental Analysis (wt.%)					*f*a*	Mw
	Pitch	PFO	C	H	N	S	O		
C100	100	-	92.8	4.2	1.2	0.4	1.4	99.9	585
C50P50	50	50	92.0	5.7	0.6	0.2	1.5	95.1	552
C33P67	33	67	92.0	6.2	0.5	0.1	1.2	92.6	526
C25P75	25	75	92.0	6.4	0.3	0.1	1.2	90.3	479
P100	-	100	90.3	7.9	1.6	0.1	0.1	80.9	420

*f*a*: Carbon aromaticity degree.

**Table 4:** Spinning results of spinnable pitch prepared by co-carbonization of purified coal tar pitch and PFO in various ratios.

Spinnable Pitch	Winding Speed (RPM)	SP		Number of Fiber Breakage (for 5 min)
		Before	After	
C100	500	250	250	Impossible
C50P50	500	250	250	5
C33P67	500	250	250	0
C25P75	500	250	255	0
P100	500	240	250	0

**Table 5:** Mechanical properties of isotropic pitch-based carbon fibers prepared by various methods and raw materials.

Isotropic Pitch-Based Carbon Fiber Samples	Average Dimeter (μm)	Tensile Strength (MPa)	Elongation (%)	Modules (GPa)	Reference
C50P50	10	870	2.21	51.5	
C33P67	9	1000	2.85	65.6	
C25P75	10	820	1.85	37.4	
Petroleum pitch-based	11	700	1.65	22.5	
	15	650	-	-	[34]
	30	210	-	22.0	[35]
	11	820	2.05	40.3	[36]
Coal tar pitch-based	14	920	-	-	[37]
	17	-	-	-	[38]
	12	786	-	-	[17]
Lignin-based	10	520	-	28.6	[39]
	33	458	0.79	59.0	[40]
	8	660	1.63	40.7	[41]
Hyper-coal-based	7	800	-	-	[19]
	8	1350	-	-	[42]

## Data Availability

The data presented in this study are available on request from the corresponding author.
